# scDLC: a deep learning framework to classify large sample single-cell RNA-seq data

**DOI:** 10.1186/s12864-022-08715-1

**Published:** 2022-07-12

**Authors:** Yan Zhou, Minjiao Peng, Bin Yang, Tiejun Tong, Baoxue Zhang, Niansheng Tang

**Affiliations:** 1 College of Mathematics and Statistics, Institute of Statistical Sciences, Shenzhen Key Laboratory of Advanced Machine Learning and Applications, Shenzhen University, Shenzhen, China; 2 Department of Mathematics, Hong Kong Baptist University, Kowloon Tong, Hong Kong; 3 School of Statistics, Capital University of Economics and Business, Beijing, China; 4 Yunnan Key Laboratory of Statistical Modeling and Data Analysis, Yunnan University, Kunming, China

**Keywords:** Single-cell RNA sequencing, Deep learning, Classifier

## Abstract

**Background:**

Using single-cell RNA sequencing (scRNA-seq) data to diagnose disease is an effective technique in medical research. Several statistical methods have been developed for the classification of RNA sequencing (RNA-seq) data, including, for example, Poisson linear discriminant analysis (PLDA), negative binomial linear discriminant analysis (NBLDA), and zero-inflated Poisson logistic discriminant analysis (ZIPLDA). Nevertheless, few existing methods perform well for large sample scRNA-seq data, in particular when the distribution assumption is also violated.

**Results:**

We propose a deep learning classifier (scDLC) for large sample scRNA-seq data, based on the long short-term memory recurrent neural networks (LSTMs). Our new scDLC does not require a prior knowledge on the data distribution, but instead, it takes into account the dependency of the most outstanding feature genes in the LSTMs model. LSTMs is a special recurrent neural network, which can learn long-term dependencies of a sequence.

**Conclusions:**

Simulation studies show that our new scDLC performs consistently better than the existing methods in a wide range of settings with large sample sizes. Four real scRNA-seq datasets are also analyzed, and they coincide with the simulation results that our new scDLC always performs the best. The code named “scDLC” is publicly available at https://github.com/scDLC-code/code.

**Supplementary Information:**

The online version contains supplementary material available at (10.1186/s12864-022-08715-1).

## Background

The development of RNA sequencing (RNA-seq) has enabled unprecedented insight into the dynamics of gene expression [1–7]. In contrast to microarray data, next-generation sequencing data improve the specificity and sensitivity of gene expression and have been increasingly popular in biological and medical research, such as detecting differentially expressed genes and identifying which type of diseases a new patient belongs to with gene expression. In recent years, a single-cell RNA-sequencing (scRNA-seq), allowing sequencing to be conducted on the level of single cells, has become another standard tool in biological and medical studies [8–12]. The scRNA-seq data analysis not only discovers new cell types, but also reveals the deep regulatory networks [13–16]. Among them, cell type identification is an important task in scRNA-seq data analysis [17]. In a general way, we identify cell types with unsupervised clustering within scRNA-seq data and then do the manual annotation based on a set of known marker genes [18]. In practice, we rarely know the number of clusters in advance, and the annotation of clusters is also somewhat subjective [19]. This may lead to bias in the analysis of the better characterised cell types. In contrast, supervised learning methods can identify the cell types more accurately and also reduce the bias associated with marker gene selection in cell type annotation.

For the classification of RNA-seq data, several statistical methods have been developed [20, 21], in particular for the bulk RNA-seq experiments. Poisson and negative binomial distributions are two most commonly used distributions to model the discrete RNA-seq data. Witten [22] assumed that the RNA-seq data follow a Poisson distribution and proposed the Poisson linear discriminant analysis (PLDA). Dong et al. [23] took into account the overdispersion of the RNA-seq data and proposed the negative binomial linear discriminant analysis (NBLDA). Note also that RNA-seq data may have excess zeros, especially when the sequence depth is not enough. Zhou et al. [24] further proposed the zero-inflated Poisson logistic discriminant analysis (ZIPLDA) with a point mass at zero when classifying RNA-seq data.

Nowadays, scRNA-seq data have been increasingly used to identify cell types and disease states for new patients. Yet to the best of our knowledge, there are still relatively few methods in the literature to classify scRNA-seq data despite the enormous potential of scRNA-seq data. Generally, low sequencing depths cause high noise levels and a large fraction of so-called “dropout” events in scRNA-seq data; and moreover, classification methods for bulk RNA-seq data may cause unacceptably large misclassification rates for scRNA-seq data. Especially for scRNA-seq data with relative large sample sizes, they may follow a more complex mixed distribution. Most existing classification methods for RNA-seq data require a certain distribution assumption, and they may fail in improving the classification accuracy for scRNA-seq data with large sample sizes. Alquicira-Hernandez et al. [25] developed a novel classification method based on singular value decomposition and a support vector machine model for scRNA-seq data. Zhao et al. [26] reviewed the existed classification tools for scRNA-seq data. Lin et al. [27] proposed a scClassify method by using a distance weighted kNN classifier. More recently, Wang and Li [28] proposed a scale-invariant deep-neural-network classifier (SINC) method which is based on deep neural-network (DNN) to classify scRNA-seq data. Their method provides a new way to dig more information for large sample size and also a novel thinking of scale invariant for next generation sequencing data. From another perspective, however, we note that the SINC method does not consider the dependency between the feature genes so that the settings may not be very realistic.

In this paper, we consider a deep learning classifier (scDLC) to identify cell types for large sample scRNA-seq data, which is based on the two-layer long short-term memory recurrent neural networks (LSTMs). The deep learning classifier can learn scRNA-seq data without the need of a distribution assumption. What’s more, the scDLC method considers the dependency between the feature genes in the process of classification. LSTMs [29] is a special kind of recurrent neural network which can learn long-term dependencies of a sequence. For scRNA-seq data, scDLC can automatically learn each sample of the class as a gene sequence.

Our scDLC framework for identifying cell types in scRNA-seq data can be summarized as four steps. For the first fully connected layer, the gene sequences of a sample are mapped to a larger dimension. The first step aims to enlarge the information of gene sequence and make the class difference more obvious. Second, the output of the first fully connected layer is taken as the input of the two-layer long short-term memory network layer, and the weights of all gates are estimated by network calculation in each class. Third, we reduce the output dimensions to the number of classes in the second fully connected layer. Lastly, the outputs of the second fully connected layer are transformed to a probability distribution with a softmax function. In the process of training, we compare the probability of each class to the observation and estimate the optimization parameters under the cross-entropy loss function.

To summarize the main advantages of the scDLC framework for classifying scRNA-seq data with large sample sizes, we note that scDLC is applicable to all scRNA-seq data no matter what the underlying distribution is. Moreover, scDLC has the capacity to capture the difference information of gene sequence from different classes, which is another key reason why it can perform the best compared to the existing competitors. In Methods, we propose the framework of scDLC and further describe the estimation of parameters in details. In Simulation studies, we conduct simulation studies to evaluate the performance of the new classifier and compare it with existing methods. In Application to Real Data, we apply the proposed method to analyze four real scRNA-seq datasets to demonstrate its usefulness in practice. We then conclude the paper in Discussion with some discussion and future directions.

## Results

We propose a deep learning framework (scDLC) based on the LSTMs model to classify scRNA-seq data. The details of the scDLC model have been shown in Methods. To validate the performance of proposed method, we consider simulation studies and real data analysis. All the R scripts that analysed the data have been uploaded at github, which could be accessible at https://github.com/scDLC-code/scDLC.

### Simulation studies

In this section, we evaluate the performance of the proposed scDLC method via simulation studies. To generate scRNA-seq read count data, we apply the Splatter Bioconductor package [30] that is known to be simple, reproducible and well-documented. While for comparison, we also consider seven other methods including PLDA, NBLDA, ZIPLDA, the support vector machines (SVM), scPred, scClassify and the SINC method.

#### Simulation design

In each experiment, we generate *n* samples for the training set and another *n* samples for the test set. We first consider the binary classification with *K*=2. Study 1 investigates the effect of different sample sizes for the binary classification. We fix the proportions of differentially expressed genes *DE*=0.5, the probability of excess zeros *pzero*=0.2, and consider the gene number *g*=100, 200, 300 and 400. We then compute the misclassification rates of all methods with different sample sizes ranging from 100 to 900. In Study 2, we evaluate the performance of all methods when the proportions of differentially expressed genes are 0.2, 0.3, 0.4, 0.5, 0.6 and 0.7 with fixed sample size *n*=200, 300, 400 and 500. In addition, we set the probability of excess zeros *pzero*=0.2 and the gene number *g*=100. In Study 3, we test the performance of all methods with the different probability of excess zeros, including *pzero*= 0.1, 0.2, 0.3, 0.4, 0.5 and 0.6. For other settings, we let the gene number *g*=100, the sample size *n*=200, 300, 400 and 500, and 40% of genes be differentially expressed.

For the multiple classification with *K*=3, we also conduct three studies to evaluate the performance of the different methods. In Study 4, we evaluate the effect of different sample sizes with three classes. All other parameters are kept the same as those in the binary classification except for the sample sizes. We set *n*= 300, 400, 500 and 600 for three classes in Studies 5 and 6, respectively.

#### Simulation results

**Fig. 1:**
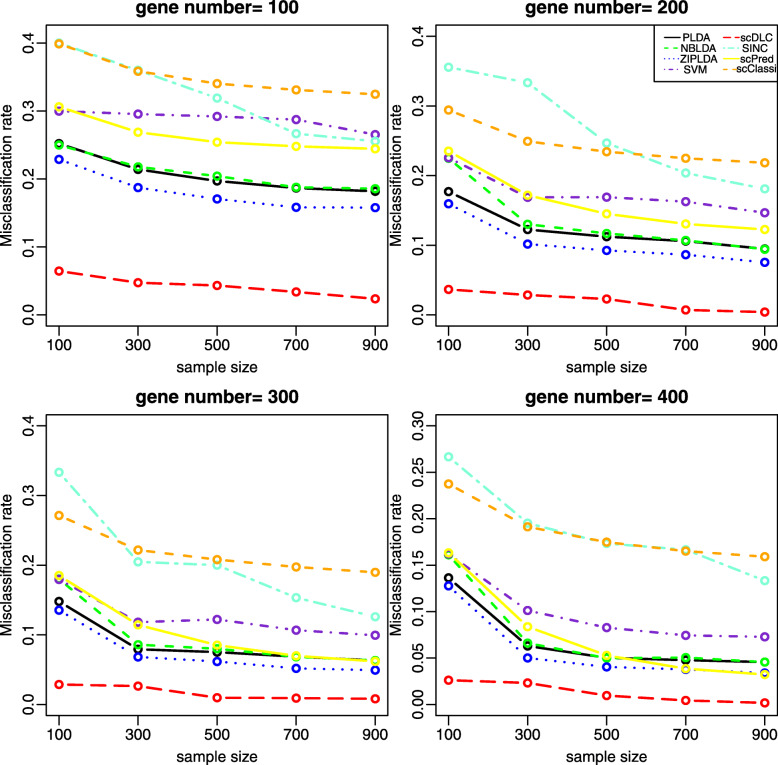
The misclassification rates of all methods with different sample sizes for two classes (Study 1). Here, *DE*=0.5 and *pzero*=0.2 for all plots. The four plots are with the gene number *g* = 100, 200, 300 or 400, respectively

**Fig. 2:**
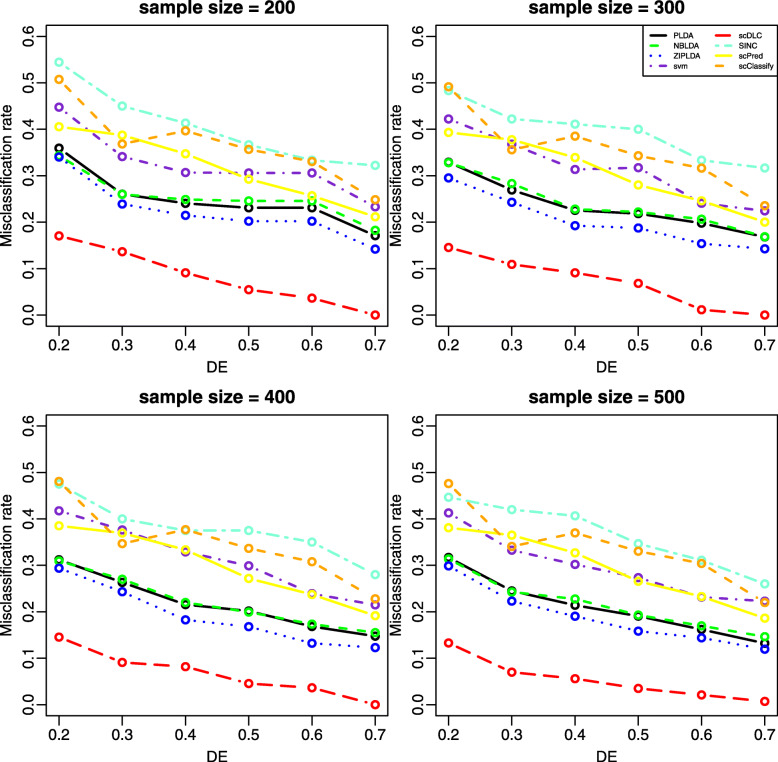
The misclassification rates of all methods with different DE rates for two classes (Study 2). Here, *g*=100 and *pzero*=0.2 for all plots. The four plots are with the sample size *n* = 200, 300, 400 or 500, respectively

With 1000 simulations for each experiment, we report the average misclassification rates for the binary classification in Figs. 1-2 and Supplementary Fig. S1, respectively. The results for the multiple classification are presented in Supplementary Figs. S2-S4. Figure 1 shows that the misclassification rates of all the considered methods decrease as the sample size increases. It is also evident that scDLC performs much better than the other methods in all cases. Figure 2 shows that the misclassification rates of all methods are decreased with an increasing number of differentially expressed genes, and meanwhile scDLC shows its superiority over the other methods. From SupplementaryFig. S1, we note that an increasing probability of excess zeros will yield a higher misclassification rate and the proposed method again outperforms the other methods in all settings.

Supplementary Figs. S2 to S4 display the simulation results for the multiple classification with *K*=3. They coincide with the conclusions made for the binary comparison, and in particular, scDLC always performs the best. Moreover, we note that SINC does not perform well when the number of selected feature genes is small, and so it can only be recommended for large number of selected feature genes.

### Application to real data

To further evaluate the performance of the different classifiers, we also analyze six scRNA-seq datasets which are from National Center for Biotechnology Information Search database (NCBI, https://www.ncbi.nlm.nih.gov/). The six datasets are summarized in Table 1. The first dataset GSE99933 was released in Furlan et al. [31]. It is used to demonstrate that large numbers of chromaffin cells arise from peripheral glial stem cells. This dataset has two classes, including 384 samples recombining at E12.5 and 384 samples recombining at E13.5. The second dataset GSE123454 illustrates the high information content of nuclear RNA for characterization of cellular diversity in brain tissues [32]. This dataset includes 463 samples from single nuclei and 463 samples from matched single cells with measurements on 42003 genes. The third dataset GSE113069 is a testament to the diversity of subiculum pyramidal cells from the hippocampus [33]. It contains three classes, each with 345, 422, 423 samples, respectively. The fourth dataset GSE84133 Baron1 was created by Baron et al. [34], and was further analyzed by the deep-neural-network classifier SINC [28]. Baron1 contains all major cell groups from the first human donor, excluding those with less than 20 cells. It contains nine classes, each with 110, 51, 236, 872, 214, 120, 130, 70 and 92 samples, respectively. The last two datasets are large sample datasets which contain tens of thousands of cells. Specifically, the fifth dataset GSE107585 was used to reveal potential cellular targets of kidney disease [35]. It came from healthy mouse kidneys, containing total 43745 cells for all fifteen classes, each with 26482, 8544, 1729, 1581, 1308, 1001, 870, 643, 549, 313, 235, 228, 110, 78 and 74 samples, respectively. The sixth dataset PBMC can be downloaded from the Single Cell Portal with accession numbers SCP424 in https://singlecell.broadinstitute.org/single_cell/study/SCP424/single-cell-comparison-pbmc-data [36]. The dataset was from human organism that contains 31021 cells for all thirteen classes, each with 7805, 6437, 4391, 3529, 2881, 2197, 1466, 908, 620, 372, 203, 149, 52, and 11 samples, respectively.

**Table 1:** Details of the four scRNA-seq datasets

Datasets	Sample size	No. of classes	No. of genes
GSE99933	768	2	23420
GSE123454	926	2	42003
GSE113069	1190	3	23218
GSE84133(Baron1)	1895	9	20126
GSE107585	43745	15	16272
PBMC	31021	13	29669

**Fig. 3:**
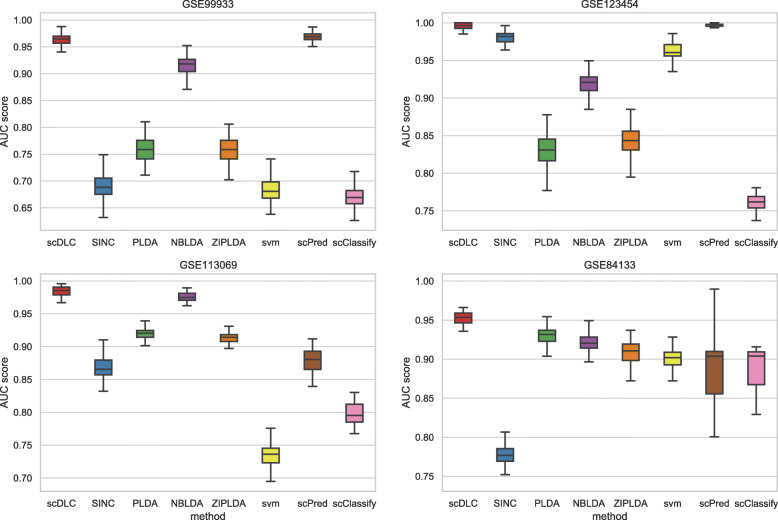
The classification experiment is repeated 100 times for four real datasets, and the AUC score results obtained are plotted as the boxplot

We assess the performance of our proposed scDLC method with seven baseline methods, including three traditional classifiers based on the Bayesian, scPred, scClassify, SVM and SINC methods. We apply the AUC score, which is the area surrounded by the coordinate axis under the ROC curve [37], to measure the performance of the classifiers. We randomly draw 40 to 450 of the samples to build the training set, and regard the rest as the test set. In real data, the majority of genes are not differentially expressed and they are irrelevant for class distinction. For example, we observe in Fig. 2 that the large rate of feature genes for class distinction will improve the accuracy of the classifiers. Thus to improve the rate of feature genes, we follow Zhou et al. [24] to select the top *p* feature genes from the training set using the BW method. Specifically for the *j*th gene, the BW value is defined as the ratio of the sum of squares between groups (BSS) to that within groups (WSS) as follows:

1 $$  \text{BW}(j) =\frac{\sum^{K}_{k=1}\sum^{n_{k}}_{i=1}({\bar{\boldsymbol{x}}}_{k.j}-{\bar{\boldsymbol{x}}}_{..j})^{2}}{\sum^{K}_{k=1}\sum^{n_{k}}_{i=1}({\boldsymbol{x}}_{kij}-{\bar{\boldsymbol{x}}}_{..j})^{2}},  $$

where ${\bar {\boldsymbol {x}}}_{..j}$$={1\over K}\sum _{k=1}^{K} {1\over n_{k}}\sum _{i=1}^{n_{k}} x_{kij}$ is the averaged expression values across all samples, ${\bar {\boldsymbol {x}}}_{k.j}$$={1\over n_{k}}\sum _{i=1}^{n_{k}} x_{kij}$ is the averaged expression value across samples belonging to class *k*, and *K* is the number of classes. Moreover, without loss of generality, we retain the top *p*=100 feature genes from each simulation as the inputs of the first layer of scDLC. We further repeat all the experiments 100 times and calculate the average AUC scores. We also present their respective boxplots in Fig. 3 with the AUC scores. From the boxplots, it is evident that our proposed scDLC outperforms the baseline methods for all four datasets.

**Fig. 4:**
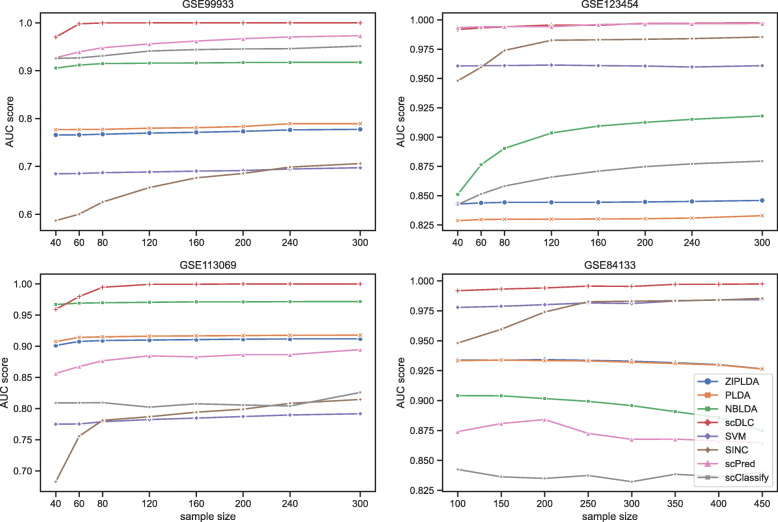
The AUC scores of all classifiers with different training sample sizes for the first four real datasets with small sample size

**Fig. 5:**
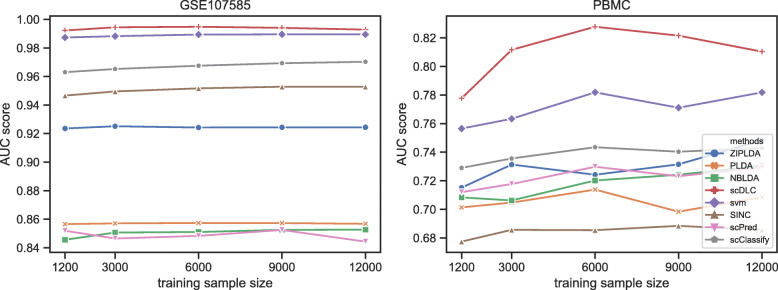
The AUC scores of all classifiers with different training sample sizes for the last two real datasets with large sample

Next, we compare the performance of all classifiers with different sizes of training samples. Figure 4 shows the AUC scores of the eight methods with different sizes of training samples for the first four real datasets with small sample size. The number of feature genes is fixed at 100 and the training sample size varies from 40 to 450. From Fig. 4, although the AUC scores of the proposed method are not outstanding when the training sample size is smaller than 50, it is still the best classifier on the whole. In particular, when the training sample size is larger than 100, our scDLC is consistently better than all other methods. As shown in Figs. 3 and 4, ScPred is comparable to scDLC for GSE99933 and GSE123454 datasets and they are both better than the other methods, which contain only two cell types. Figure 5 shows the AUC scores of the eight methods with different sizes of training samples for the last two real datasets with large sample. The number of feature genes is fixed at 100 and the training sample size varies from 1200 to 12000. From Fig. 5, the proposed method outperforms the exiting methods for large training sample in the two real datasets. The AUC scores of SVM are less than those of our scDLC but much higher than the other methods.

**Fig. 6:**
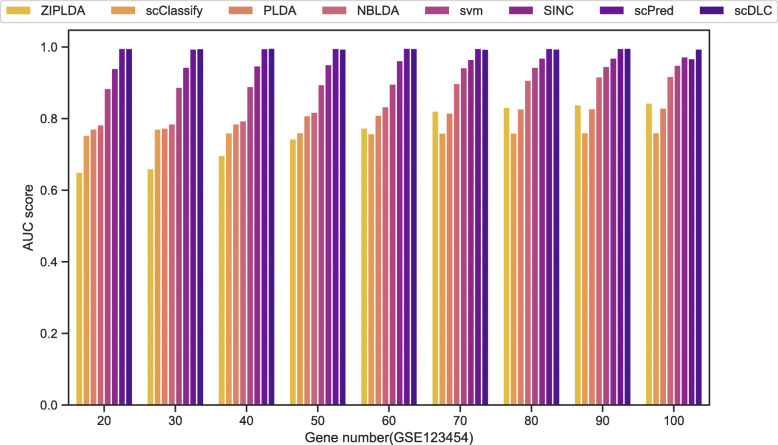
The AUC scores of all classifiers with different feature gene number for GSE123454 dataset

Finally, we consider the performance of each classifier under different selected feature genes. Specifically, we use 70% of the dataset as the training set and the rest as the test set. According to the degree of differential expression, the top 20 to 100 genes are selected to test the performance of each classification method. Figure 6 and Supplementary Figs. S5-S7 show the AUC scores of the eight methods with different selected feature genes. For the GSE123454 and GSE99933 datasets in Fig. 6 and Supplementary Fig. S5, the scPred method is comparable to the scDLC method and much better than the other methods. However, NBLDA is comparable to the scDLC method in Supplementary Fig. S7. In Supplementary Figs.S6 and S7, we observe a similar result that the scDLC method outperforms the other methods in the GSE84133 and GSE113069 datasets. The four Figures show that the comparison results of the classifiers are relatively consistent under different choices of the selected genes and the proportion. Finally, it is noteworthy that the AUC scores of scDLC are not affected much by the number of feature genes.

## Discussion

The single-cell RNA sequencing (scRNA-seq) technology has been increasingly used in molecular diagnosis of clinical diseases. In this paper, we proposed a deep learning framework with two layers of LSTMs, namely scDLC, to classify large sample scRNA-seq data. The innovation of scDLC is mainly manifested in two aspects. Firstly, compared to the existing discriminant rules, our new method does not require a distribution assumption so that it can be widely applied in practice. Secondly, our scDLC also amplifies the features of the selected genes through the first fully connected layer. It is thus beneficial to improve the classification accuracy and stability of the model, and meanwhile our scDLC can be trained with less computer resource using only the top selected feature genes.

To evaluate the performance of our new classifier, we considered both the binary classification and the multiple classification. Simulation results show that our deep learning method can sufficiently capture the difference information of classes in gene sequences, and that it performs much better than, or at least as well as, the existing competitors in a wide range of settings with large sample sizes. We also analyzed six real scRNA-seq datasets, including both small and large sample sizes, and they all support that our new scDLC always performs the best.

As a future work, we will study from the network structure level why scDLC can efficiently capture class differences from gene sequences, and we expect that understanding the mechanism can bring deep insights to gene expression and regulation. Moreover, it can also be interesting to extend deep learning techniques to conduct in-depth research in precision medicine such as neonatal genetic disease-related gene screening.

## Methods

We first review the framework of long short-term memory recurrent neural networks (LSTMs), and then introduce a new workflow of the deep learning classifier (scDLC) for large sample size scRNA-seq data.

**Fig. 7:**
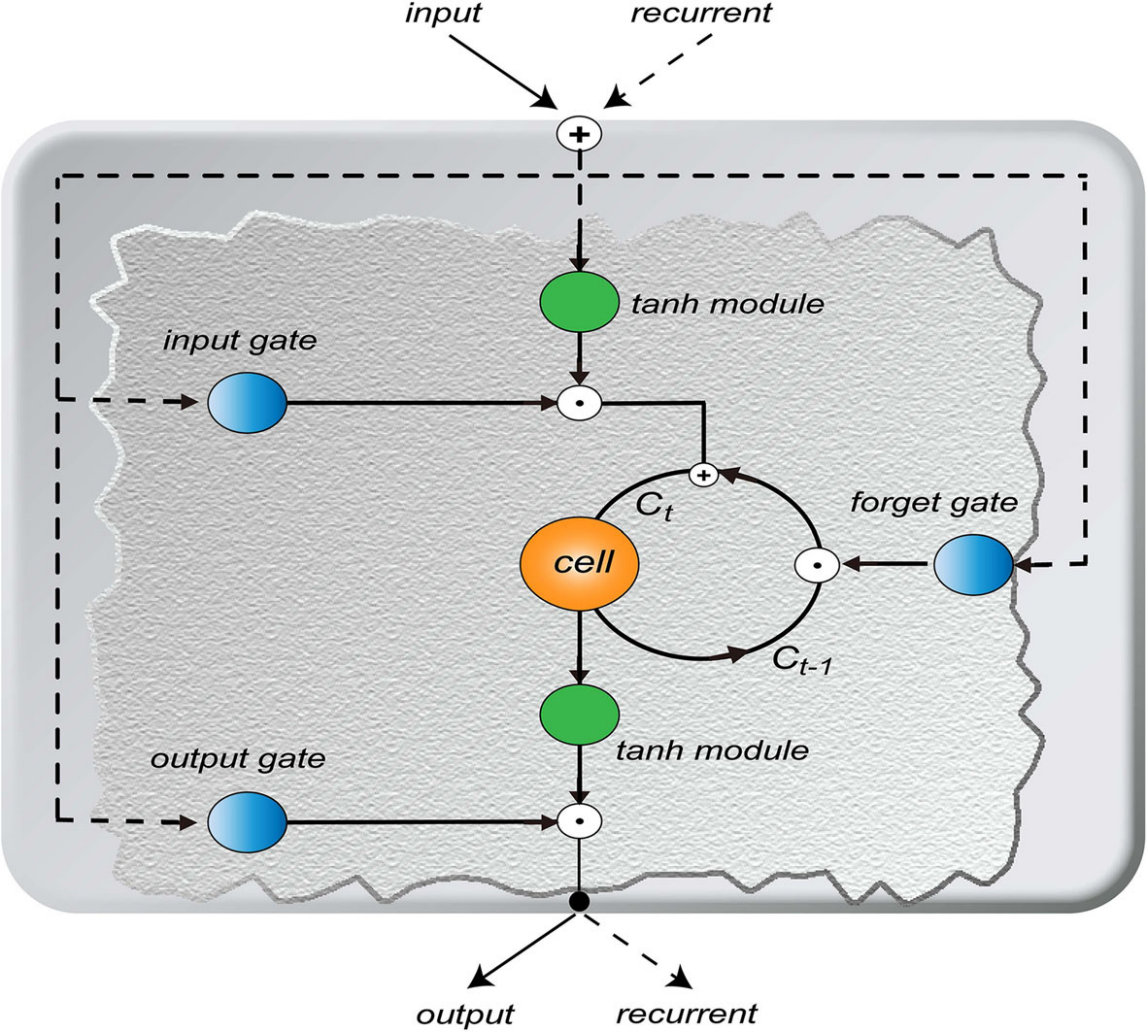
The framework of LSTMs. It consists of three gates (input gate, forget gate and output gate) and two tanh modules. The data of the current time and the cell state of the previous time are combined and input into the LSTMs. After a series of nonlinear transformations, the current cell state and output are obtained

Hochreiter and Schmidhuber [29] proposed a recurrent neural network with long short-term memory network. This network has a great performance to solve the sequential data related learning problem. LSTMs can effectively capture both short-term and long-term time dependence. Sak et al. [38] showed that the long short-term memory network is effective for acoustic modeling. Marchi et al. [39] proposed a bidirectional LSTMs for audio onset detection. Due to the gate mechanism, LSTMs solves the problem of gradient vanishing which cannot be overcomed by the simple recurrent neural network. The early LSTMs was refined and popularized by many people in the following work. The structure of this model was further improved by Graves et al. [40] based on the previous research [41, 42]. The core idea of the LSTMs is several non-linear gating units that control information retention and forgetting, as well as a memory cell that can maintain its state over time. As shown in Fig. 7, it includes a single cell, two tanh activation blocks and three gates (input gate, forget gate, output gate). The input gate controls the input information and whether the input will be read. The forget gate controls the internal state information and whether the current cell value is forgotten. The output gate controls the output information and whether new cell values are output. The input of the three gates is the output of the previous time and the input of the current time. The activation function of three gates is the sigmoid function. Let *x*_*t*_,*h*_*t*_ and *C*_*t*_ denote the input value, the output value and the cell state at time *t*, respectively. Let *b* denote the bias term, and *W* denote the weight matrix. Let also *f*, *i* and *o* denote the forget gate, the input gate and the output gate, respectively. The recurrent process of LSTMs can be expressed as follows:

2 $$ \begin{aligned} f_{t}&=\sigma(W_{f}\cdot[h_{t-1},x_{t}]+b_{f})\\ i_{t}&=\sigma(W_{i}\cdot[h_{t-1},x_{t}]+b_{i})\\ \tilde{C}_{t}&=tanh(W_{C}\cdot[h_{t-1},x_{t}]+b_{C})\\ C_{t}&=f_{t}*C_{t-1}+i_{t}*\tilde{C}_{t}\\ o_{t}&=\sigma(W_{o}\cdot[h_{t-1},x_{t}]+b_{o})\\ h_{t}&=o_{t}*tanh(C_{t}), \end{aligned}  $$

where $\tilde {C}_{t}$ is a vector of new candidate values, $\sigma (z)=\frac {1}{1+e^{-z}}$ is the sigmoid function, and $tanh(z)=\frac {e^{z}-e^{-z}}{e^{z}+e^{-z}}$ is the tanh function. In addition, “.” represents the matrix multiplication and “*” represents the multiplication with scalars.

### Deep learning classifier for scRNA-seq data

**Fig. 8:**
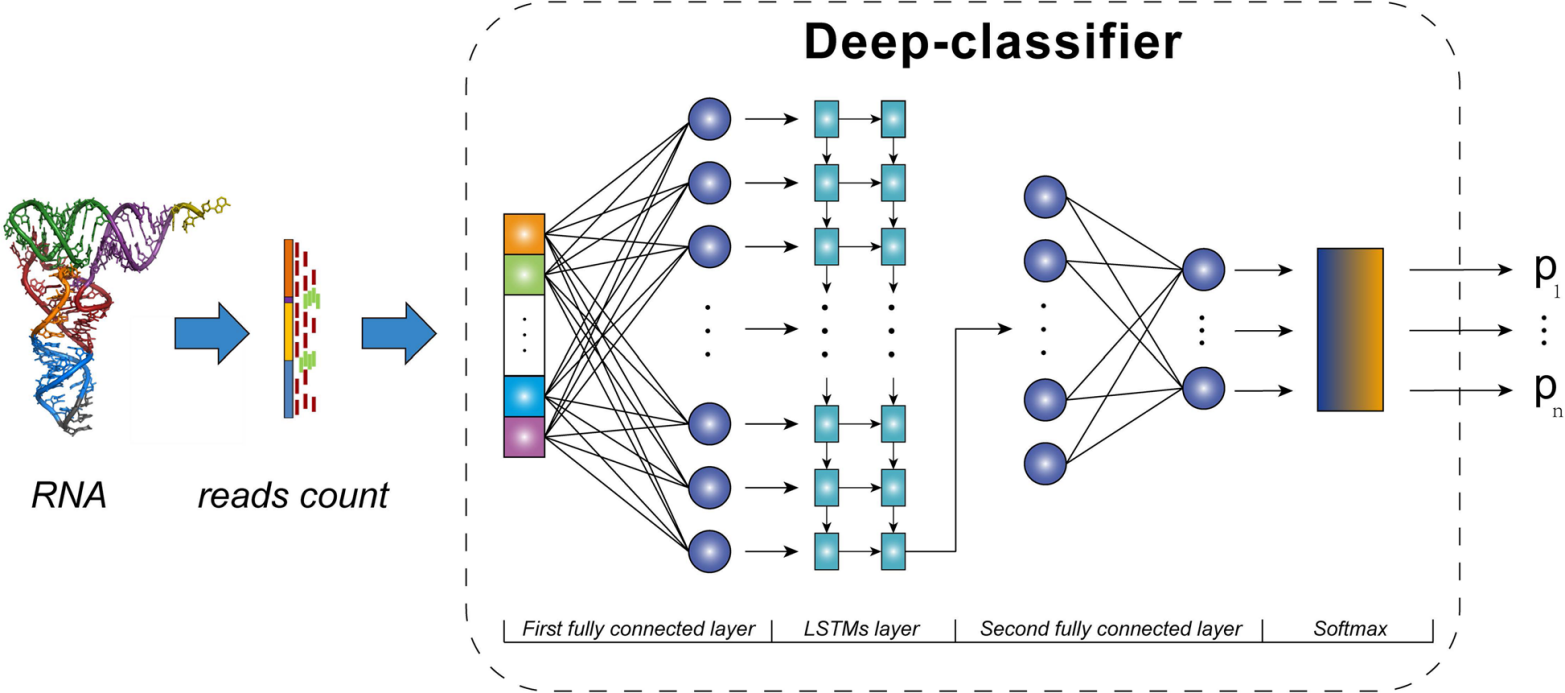
The framework of scDLC with three layers. The first layer and the third layer are two fully connected layers, and the middle layer is an LSTMs layer that consists of two long short-term memory network sub layers. A softmax layer is connected at the end to map the output of the classifier to a probability distribution

The scDLC framework is shown in Fig. 8, which includes two fully connected layers and a two-layer LSTMs. The fully connected layers are located at the first layer and the last layer, respectively. After the model training, it results in a scRNA-seq data classifier. Inputting a gene sequence sample into scDLC, the probability that the gene sequence sample belongs to each class will be obtained. Finally, we identify which class the sample belongs to based on the probability vector.

**Fully connected layers**: Each node of the fully connected layer is connected to all nodes of the previous layer. It can synthesize the extracted features through the rectified linear unit (*ReLU*) activation function. The function of the first fully connected layer in scDLC is to amplify the information of the gene sequence and make the class difference more obvious. This layer can greatly improve the accuracy of discrimination. The *ReLU* activation function is

3 $$ a=max(0,Wx+b),  $$

where *x* is the input vector, *W* is the weight matrix, *b* is the bias vector, and *a* is the activation vector which is the output of the fully connected layer. Using the *ReLU* activation function in the network can make the classifier perform better. At the end of the model, we map the output of the second fully connected layer to the probability distribution of the class through a *softmax* function as

4 $$ softmax(y_{c})=\frac{e^{y_{c}}}{\sum^{M}_{j=1}e^{y_{j}}},  $$

where *M* is the number of classes.

**LSTMs layer**: In the LSTMs layer, we take two LSTMs sublayers to learn data. The horizontal connection between sublayers means that the output *h* of the first sublayer is entered into the second sublayer as input. The vertical connection means that the cell state *C* of the previous time is transferred to the next time in the same sublayer. The output of this layer will be used as the input to the second fully connected layer. The forward recursions of this layer refer to the formulas in (Fig. 7).

The trainable parameters (all weights and biases) in this deep model are denoted as *θ*. The partial derivatives *∂L*/*∂θ* of the loss function *L* with respect to any trainable parameter in the network can be calculated by the back propagation algorithm [43]. We further take the cross entropy as the loss function since it can well describe the difference between the true probability distribution and the predicted probability distribution. To be specific, we define the loss function as

5 $$ L=-\frac{1}{N}\sum^{N}_{i=1}\sum^{M}_{c=1}y_{ic}log(p_{ic}),  $$

where *N* is the sample size, *M* is the number of classes, *y*_*ic*_ is an indication variable which is 1 if class *c* is the same as the class of the sample or otherwise 0, and *p*_*ic*_ represents the prediction probability that sample *i* belongs to class *c*.

The gradient descent method is a widely used optimization algorithm in machine learning. We use a mini-batch gradient descent algorithm (MBGD) [44] to train our model. For a set of training samples, MBGD does not use all the training samples to calculate the real gradient of the target, but instead calculates the gradient of a small batch samples. We then minimize the loss function by updating the trainable parameter *θ*. According to the MBGD algorithm, the rule for updating is as follows:

6 $$ \theta\gets\theta-\eta\frac{\partial L}{\partial \theta},  $$

where *η* is the learning rate. In order to avoid fluctuation in the later stage of training, we further set the learning rate decay exponentially during the training. That is

7 $$ \tilde{\eta}=\eta e^{\gamma/s},  $$

where $\tilde {\eta }$ is the learning rate after decay, *γ* is the decay rate, and *s* is the global step. The exponential-decay learning rate means that the learning rate is correlated with the number of training times, and it will decline exponentially with the increase of training times. Here, *r* is the decay rate, *s* is the global step, the maximum learning rate is set to *max*_*lr*=0.005, the minimum learning rate is set to *min*_*lr*=0.001, epoch is the training times, *x* is the sample size in the total training set, *batch*_*size* represents the sample size in a batch, then decay rate is computed with *r*=*log*(*max*_*lr*/*min*_*lr*)/(*epoch*∗*x*/*batch*_*size*). Then the learning rate after decay can be obtained according to the calculated *decay*_*rate*.

### Hyperparameter settings

To implement the proposed scDLC, it is further needed to determine the hyperparameters in the model. Note that the hyperparameters are the configuration outside the model, and their values cannot be estimated from the data. Appropriate hyperparameters can greatly improve the performance of the model. According to the test of different hyperparameter combinations, we set the following parameters that can yield a good performance for the classification.**hidden size =64**: The parameter represents the size of the hidden state of LSTMs and we set it as 64.**batch size =11**: For the number of samples in a batch, we randomly choose 11 samples throughout the simulations.**grad clip =5**: To stabilize the network in the process of training, we set the threshold as 5 for the gradient to control the weight update within a certain range. **train keep prob =0.3**: To prevent overfitting, we let the train keep probability equal to 0.3, which means that only 30% of the information will be used in the next time. **initial learning rate =0.005**: For the appropriate learning rate that can make the objective function converge to a local minimum at a suitable time, we set the initial learning rate as 0.005. Since the learning rate will decline with training, we further set the minimum learning rate as 0.001.

## Supplementary Information

**Additional file 1** Supplementary figures and tables. This file contains related figures and tables for simulated and real datasets.

## Data Availability

The datasets are from National Center for Biotechnology Information Search database (NCBI, https://www.ncbi.nlm.nih.gov/). The first dataset GSE99933 was released in [31]. The second dataset GSE123454 illustrates the high information content of nuclear RNA for characterization of cellular diversity in brain tissues [32]. The third dataset GSE113069 is a testament to the diversity of subiculum pyramidal cells from the hippocampus [33]. The fourth dataset GSE84133 Baron1 was created by [34]. The fifth dataset GSE107585 was released in [35]. The sixth dataset PBMC can be downloaded from the Single Cell Portal with accession numbers SCP424 [36]. All the R scripts that analysed the data are available at https://github.com/scDLC-code/scDLC. Additional supporting Figures and Tables are included as Additional files.

## Abbreviations

scDLC: Deep learning classifier for large sample scRNA-seq data
LSTMs: Long short-term memory recurrent neural networks
scRNA-seq: Single-cell RNA sequencing
FDR: False discovery rate
ROC: Receiver operating characteristic
AUC: Area under the curve
