# Supplementary material for: scDLC: a deep learning framework to classify large sample single-cell RNA-seq data
Source: BMC Genomics. 2022 Jul 12;23:504. doi: 10.1186/s12864-022-08715-1 (PMC9281153; doi:10.1186/s12864-022-08715-1)
Supplement: Supplementary file 1 — Additional file 1 Supplementary figures and tables. This file contains related figures and tables for simulated and real datasets. [file 12864_2022_8715_MOESM1_ESM.pdf]

Supplemental materials for "scDLC: a deep learning framework to classify large sample single-cell RNA-seq data"

Yan Zhou[1], Minjiao Peng[1], Bin Yang[1], Tiejun Tong[2], Baoxue Zhang[3] and Niansheng Tang[4]

[1] *College of Mathematics and Statistics, Institute of Statistical Sciences, Shenzhen Key Laboratory of Advanced Machine Learning and Applications, Shenzhen University, Shenzhen, China;* [2] *Department of Mathematics, Hong Kong Baptist University, Hong Kong;* [3]*School of Statistics, Capital University of Economics and Business, Beijing, China;* [4]*Yunnan Key Laboratory of Statistical Modeling and Data Analysis, Yunnan University, Kunming, China*

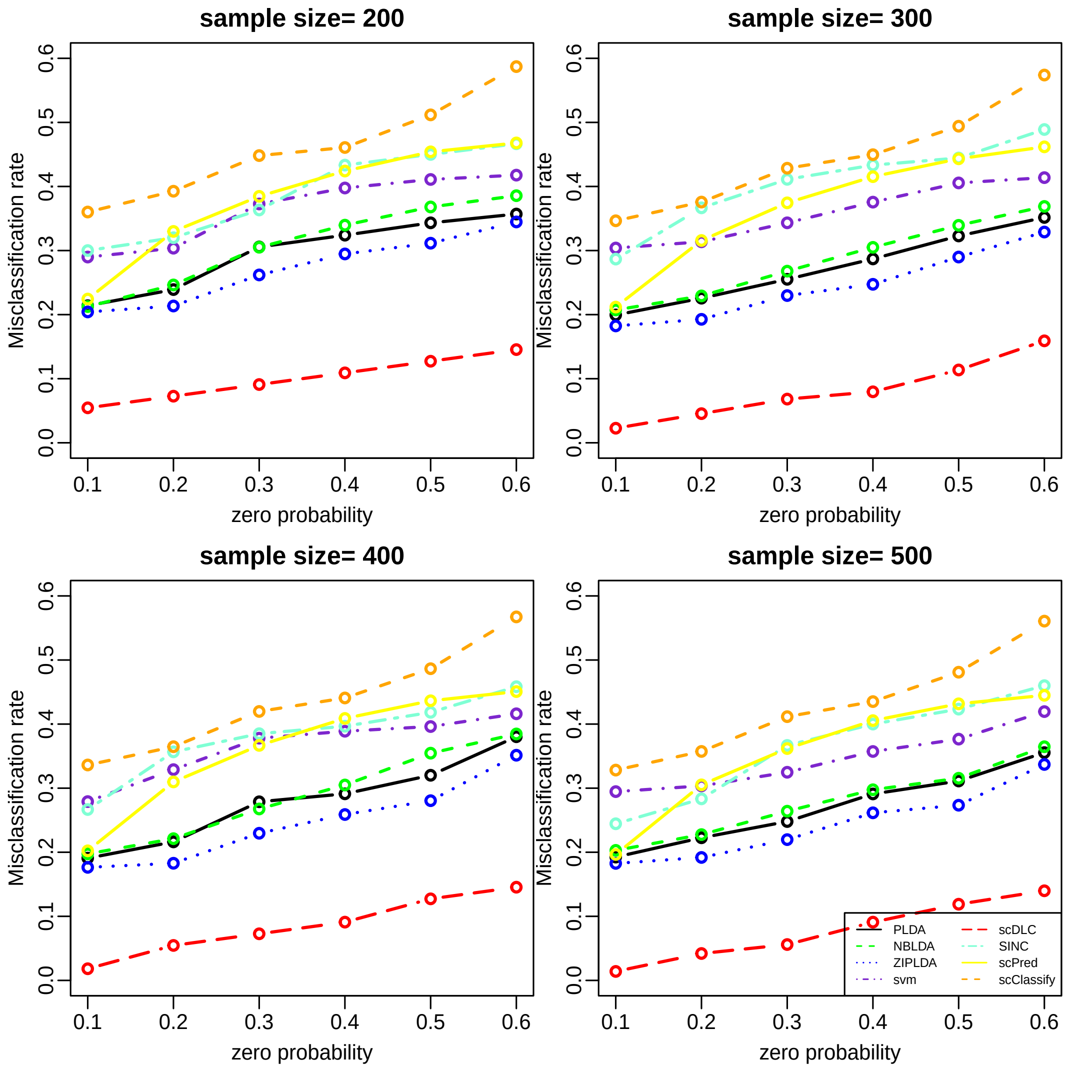

Figure S1: The misclassification rates of all methods with different zero probabilities for two classes (Study 3). Here, $g = 100$ and $DE = 0.4$ for all plots. The four plots are with the sample size $n = 200$, 300, 400 or 500, respectively.

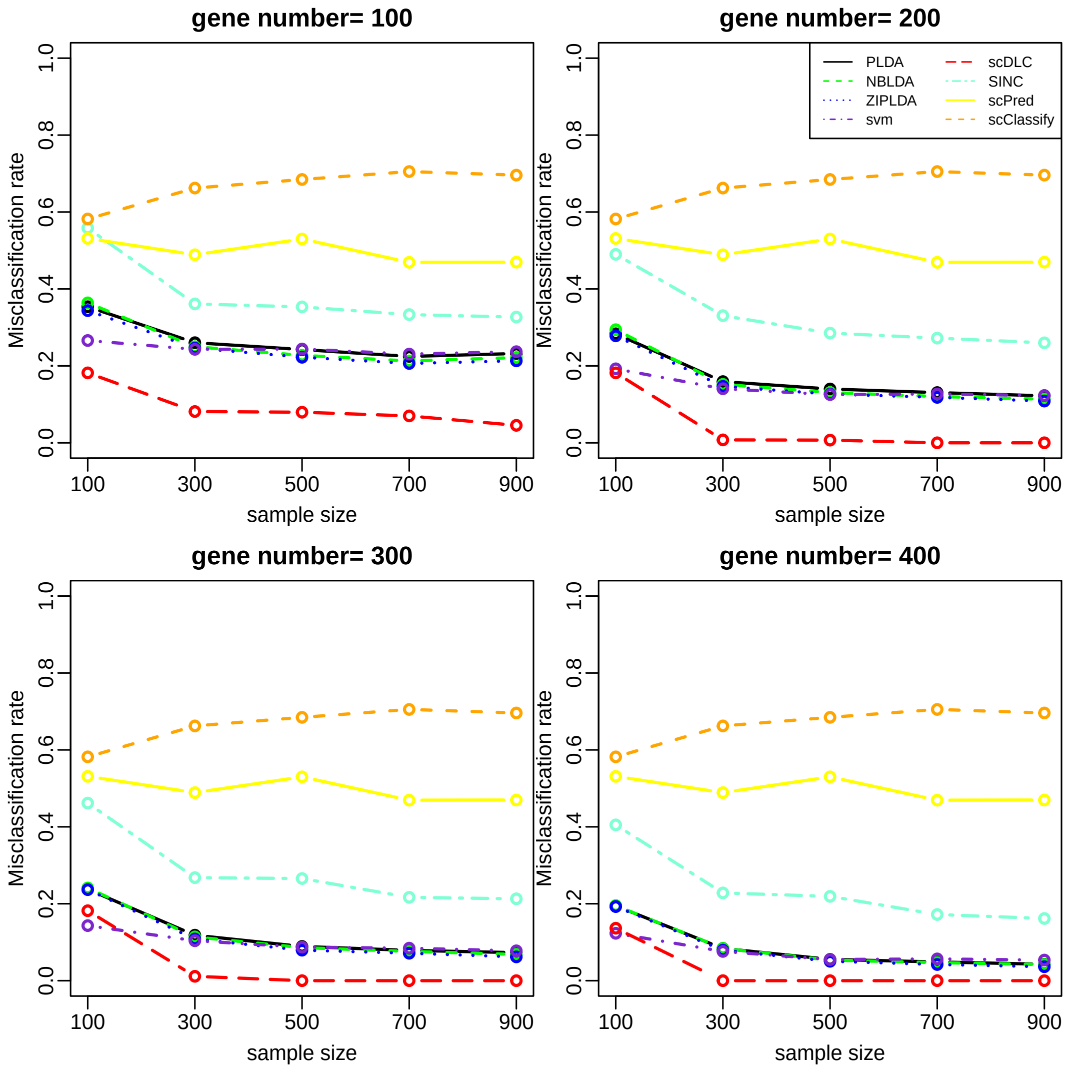

Figure S2: The misclassification rates of all methods with different sample size for three classes. Fix the gene number = 100, 200, 300 and 400 in the four sub-graphs.

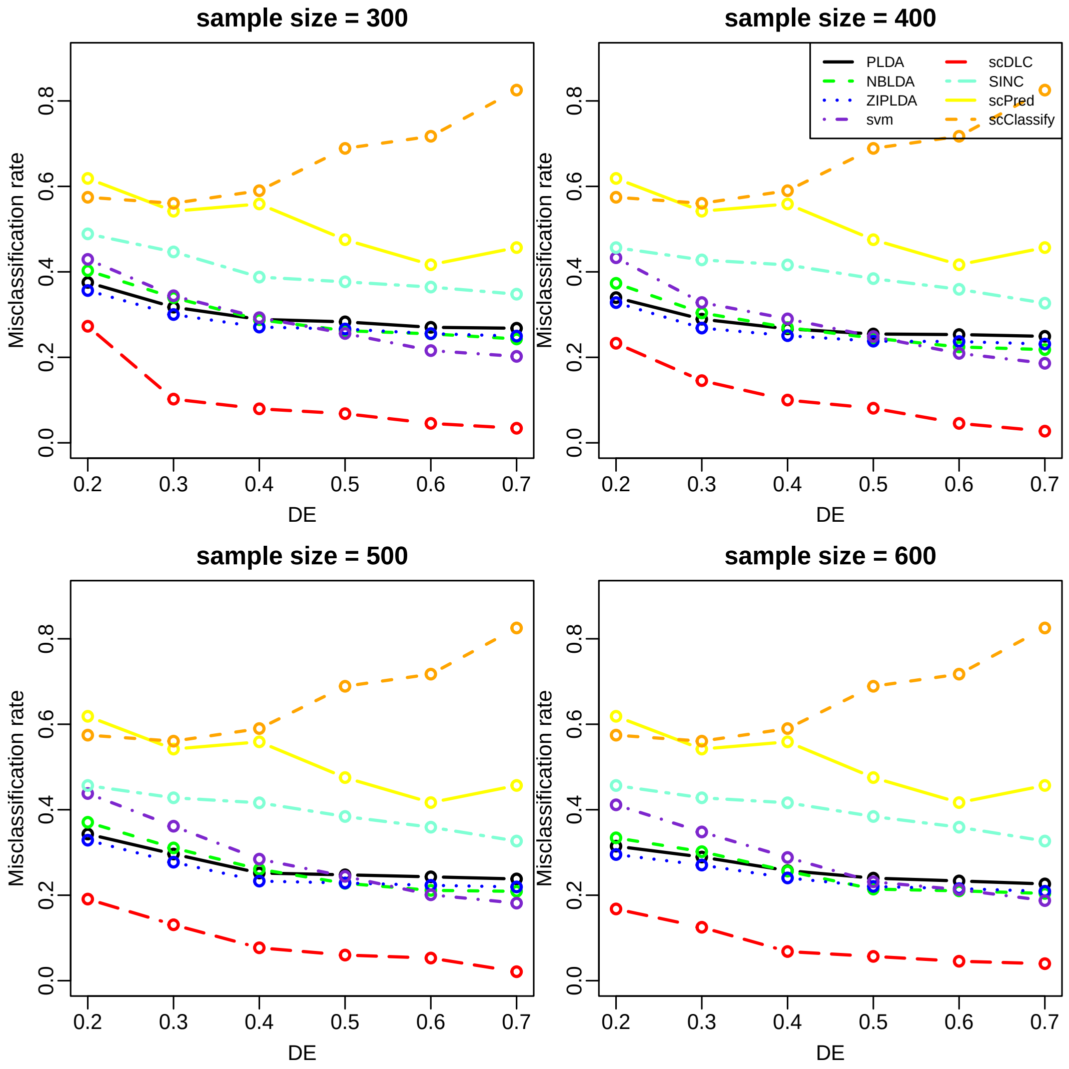

Figure S3: The misclassification rates of all methods with different DE rates for three classes. Fix the sample size = 300, 400, 500 and 600 in the four sub-graphs.

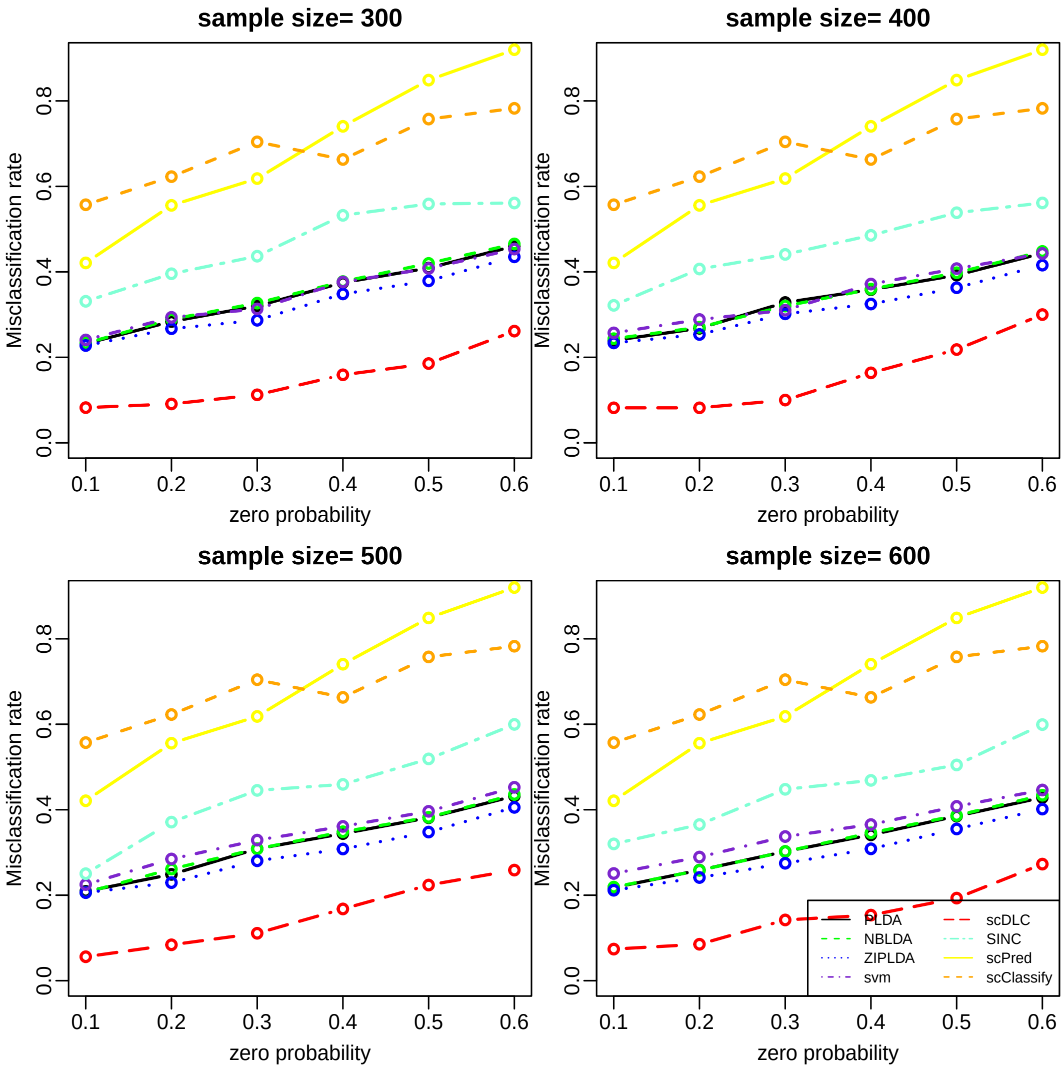

Figure S4: The misclassification rates of all methods with different zero probabilities for three classes. Fix the sample size = 300, 400, 500 and 600 in the four sub-graphs.

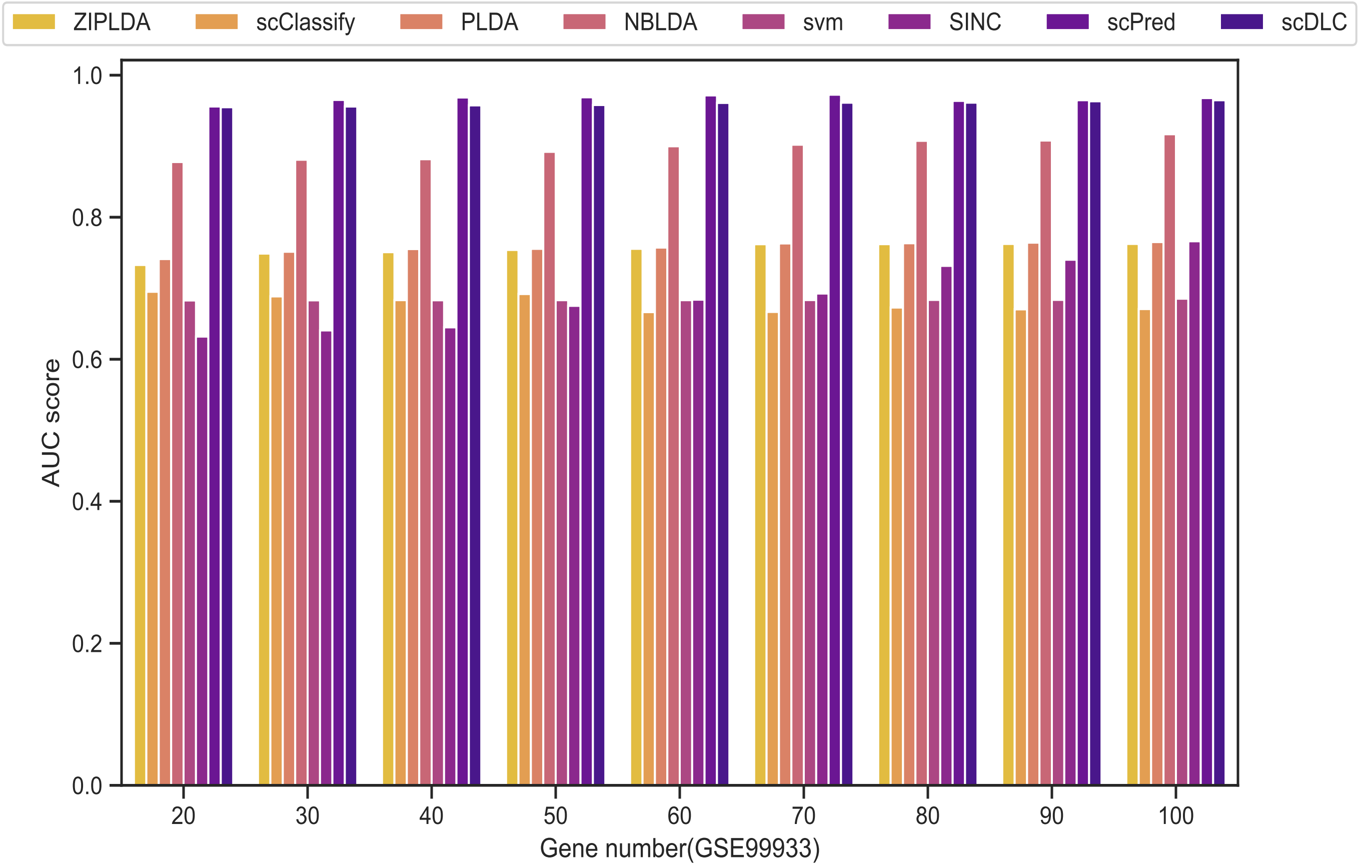

Figure S5: The AUC scores of all methods with different gene number for GSE99933 dataset. 70% of the datasets is used as a training set and the rest as a test set.

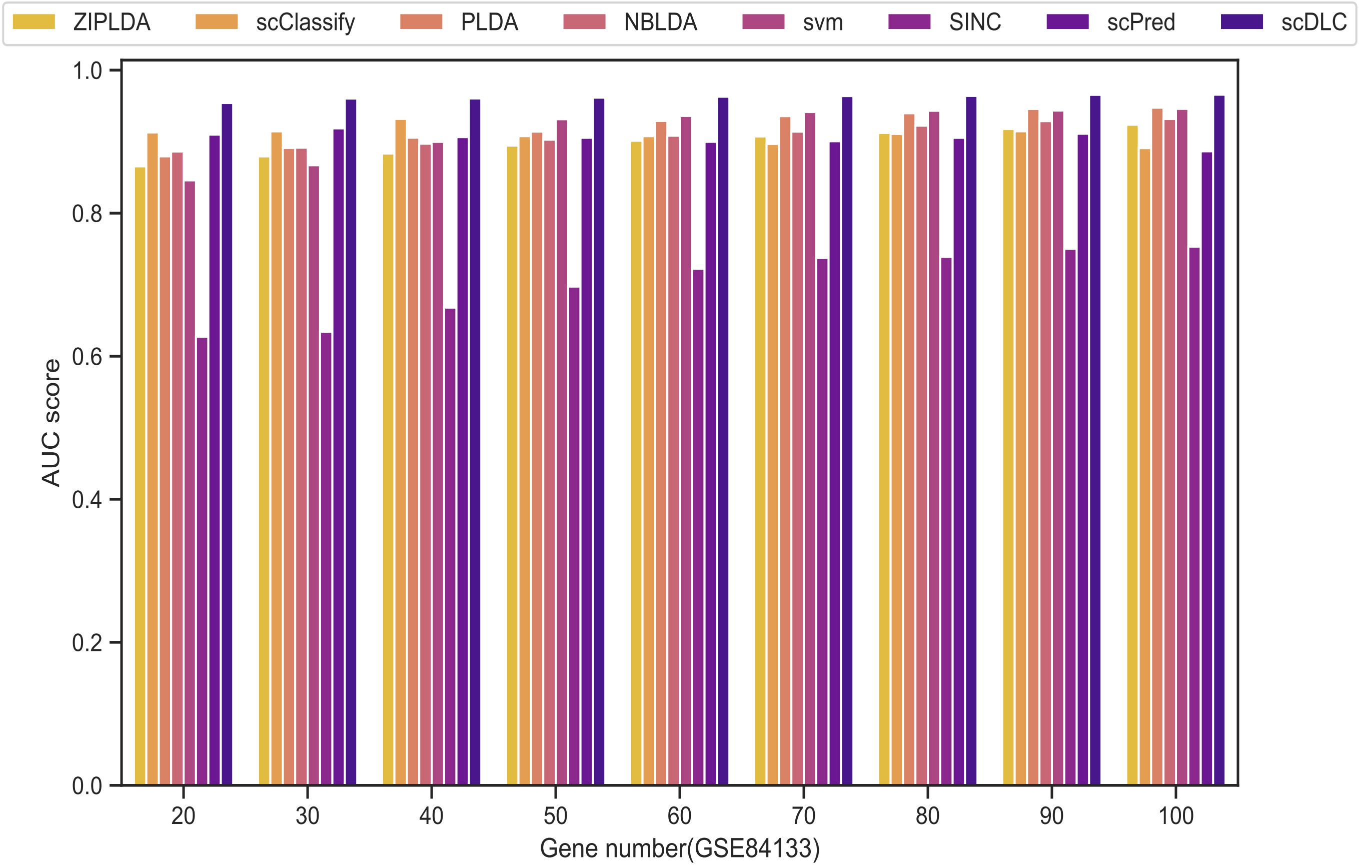

Figure S6: The AUC scores of all methods with different gene number for GSE113069 dataset. 70% of the datasets is used as a training set and the rest as a test set.

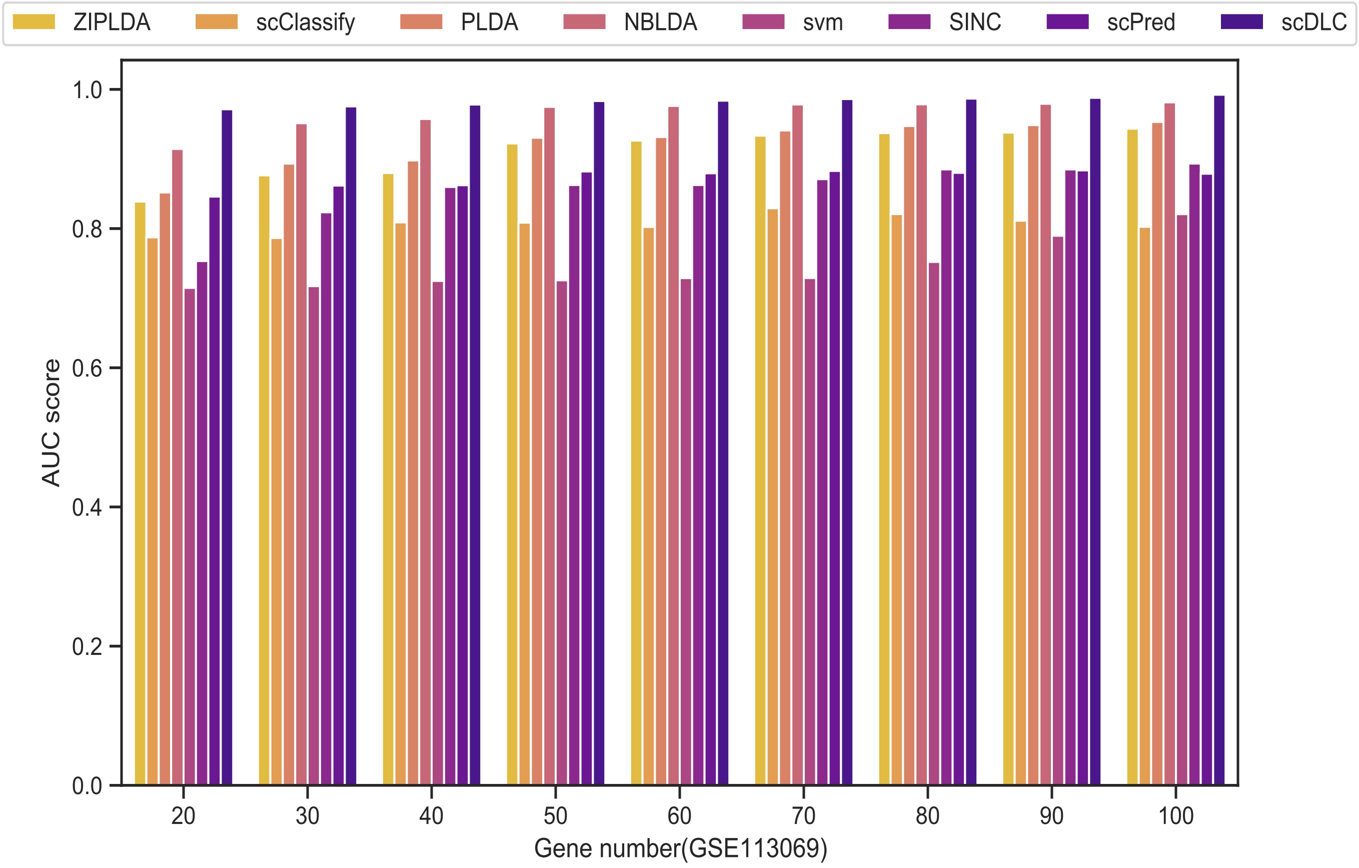

Figure S7: The AUC scores of all methods with different gene number for GSE84133 dataset. 70% of the datasets is used as a training set and the rest as a test set.
